# Construction of SARS-CoV-2 Virus-Like Particles by Mammalian Expression System

**DOI:** 10.3389/fbioe.2020.00862

**Published:** 2020-07-30

**Authors:** Ruodan Xu, Mingfei Shi, Jing Li, Ping Song, Ning Li

**Affiliations:** ^1^Institute of Basic Theory for Chinese Medicine, China Academy of Chinese Medical Sciences, Beijing, China; ^2^Dongzhimen Hospital, Beijing University of Chinese Medicine, Beijing, China; ^3^Guang’anmen Hospital, China Academy of Chinese Medical Sciences, Beijing, China

**Keywords:** COVID-19, SARS-CoV-2, virus-like particles, self-assemble, release

## Abstract

Virus-like particle (VLP) is a self-assembled nanostructure incorporating key viral structural proteins. VLP resembles molecular and morphological features of authentic viruses but is non-infectious and non-replicating due to lack of genetic materials. Successful applications of VLP has been shown in vaccinological and virological research. As an accessibly safe and relevant substitute of naturally pathogenic viruses, the construction of SARS-CoV-2 VLPs is much in demand in the ongoing fight against 2019 Coronavirus disease (COVID-19) pandemics. In the current study, using mammalian expression system, which is advantageous in maintaining correct protein glycosylation patterns, we efficiently constructed SARS-CoV-2 VLPs. We showed that among four SARS-CoV-2 structural proteins, expression of membrane protein (M) and small envelope protein (E) are essential for efficient formation and release of SARS-CoV-2 VLPs. Moreover, the corona-like structure presented in SARS-CoV-2 VLPs from Vero E6 cells is more stable and unified, as compared to those from HEK-293T cells. Our data demonstrate that SARS-CoV-2 VLPs possess molecular and morphological properties of native virion particles, which endow such VLPs with a promising vaccine candidate and a powerful tool for the research of SARS-CoV-2.

## Introduction

The ongoing pandemic of 2019 Coronavirus disease (COVID-19) is caused by the severe acute respiratory syndrome coronavirus 2 (SARS-CoV-2), a newly emergent member of *Coronaviridae* family (Coronaviridae Study Group of the International Committee on Taxonomy of Viruses, 2020; Wu et al., 2020). Though sharing 79.6% genetic sequence (Zhou et al., 2020), SARS-CoV-2 is apparently less pathogenic but more contagious as compared to SARS-CoV, which belongs to the same virus family (Oberemok et al., 2020). In the months since the COVID-19 rose from a regional crisis to a global threat, considerable research efforts testing possible treatments have been intensively exerted, however, until recently, there is still no medication and vaccines available for COVID-19 (Lythgoe and Middleton, 2020). Moreover, at molecular level, despite the most readily accessible genetic and structural deciphering, viral information of SARS-CoV-2 is far from being satisfactorily understood (Rabi et al., 2020). The lag in pharmacological and virological studies are to some extent due to the limited access to SARS-CoV-2, which requires biosafety level 3 (BSL-3) facilities (Li et al., 2005). Considering that in addition to respiratory involvement, SARS-CoV-2 can be detected in multiple organs (Wang T. et al., 2020), the interdisciplinary collaborations with virologists should be reinforced in the fight against COVID-19. On the premise of this background, development of safe experimental models as substitutes for SARS-CoV-2 are much in demand.

Pseudo-viral system, which usually utilizes adenoviral or lentiviral vectors following BSL-2 practices have been used as experimental models to study entry events of enveloped viruses, including SARS-CoV-2 (Nie et al., 2020; Ou et al., 2020). However, since pseudo-viruses generally contain only one structural proteins of the native virus, such as spike protein (S) in SARS-CoV-2, functions of other envelope proteins and the associated protein-protein interactions are prone to be overlooked when more than one structural protein are involved in the authentic viruses. The debate about pseudo-viruses has gained further prominence with many arguing that the immune responses induced by pseudo-viral system is more likely to be derived from the vector virus instead of the authentic one.

As a vaccine candidate, virus-like particle (VLP) has also been applied in the study of key processes of viral life cycle (Lopez-Macias, 2012; Low et al., 2014; Mohsen et al., 2017; Chen et al., 2020). Due to lack of genetic material that determines the pathogenicity of viruses, VLP is non-infectious and can be performed in normal laboratory settings without biosafety protection. Therefore, VLP constitutes a safe and relevant model in molecular studies of virus entry and virion egress (Li et al., 2019). Because VLP is formed by self-assembly that naturally occurs when partial or all viral structural proteins are optimally co-expressed in permissive cells, the resulting VLP is able to fully represent the original morphologic and immunogenic features of the natural virus (Noad and Roy, 2003; Grgacic and Anderson, 2006; Zhao et al., 2012; Huang et al., 2017). Technically, in constructing VLPs of more than one structural protein, cell-based expression systems can be co-transfected with a polycistronic vector or multiple monocistronic vectors. The advantage of the latter is to individually manipulate each envelope protein in order to understand the essential details that mediate VLP formation and release. A variety of expression systems have been utilized to construct VLP, including mammalian cell lines, bacteria, insect cell lines, yeast and plant cells (Buonaguro et al., 2006; Santi et al., 2006; Li et al., 2010). While the complexity of construction and applications can often be problematic for mammalian systems, the correct protein glycosylation and folding patterns that distinguish mammalian cells from other expression systems are actually of critical for viral infectivity (Shiyu Dai, 2018).

SARS-CoV-2 virions consist of four structural proteins, namely S, small envelope (E), membrane (M) and nucleocapsid (N) protein (Wang Q. et al., 2020). Since the basic requirements in the assembly of four structural proteins into SARS-CoV VLPs are not identical to those of other coronaviruses, the delineation of VLP formation of SARS-CoV-2 will provide both molecular information of SARS-CoV-2 assembly and egress, and more importantly an accessible experimental tool for the study of SARS-CoV-2. By using mammalian expression system, this study is designed to understand how to efficiently construct SARS-CoV-2 VLPs.

## Materials and Methods

### Plasmid Construction and Molecular Cloning

Human codon optimized sequences of genes encoding S, M, E and N structural proteins of SARS-CoV-2 with C-terminal FLAG tag (peptide sequence: DYKDDDDK) were synthesized by Genscript Biotechnology (Nanjing, China): the major structural S glycoprotein (Gen Bank: QHD43416.1), E protein (Gen Bank: QHD43418.1), M protein (Gen Bank: QHD43419.1) and N protein (Gen Bank: QHD43423.2). *Nhe*I and *Not*I (NEB, England BiolLabs, Beverly, MA, United States) restriction sites were placed at 5′ and 3′ ends, respectively. The four genes were cloned into the double *Nhe*I and *Not*I restriction sites of the expression vector pcDNA3.1. Then the transformation experiments were performed with chemically competent cells DH5α (TransGen Biotechnology, Beijing, China) using the heat shock method in the water bath at 42°C for 1 min, followed by shaking at 37°C for 45 min. After centrifugation at 2,800 × *g* for 3 min, the transformed cells were plated on LB plates containing 50 μg/ml ampicillin and the plates were inverted and incubated at 37°C overnight. The resistant single colony was picked and amplified in LB medium. The correct orientation of the insertions was examined by restriction enzyme analysis and the open reading frames of recombinant plasmids were verified by DNA sequencing.

### Cell Culture

HEK-293T human embryonic kidney cell line and Vero E6 African green Monkey kidney cell line were purchased from the American Type Culture Collection (ATCC, Manassas, VA, United States) and cultured in Dulbecco’s modified essential medium (DMEM) from Gibco (Carlsbad, CA, United States). The cell culture medium contained 10% fetal bovine serum (FBS, Gibco), penicillin (100 U/ml) and streptomycin (100 μg/ml) and cells were maintained with 5% CO_2_ at 37°C.

### Synthesis and Purification of VLPs

**FIGURE 1:**
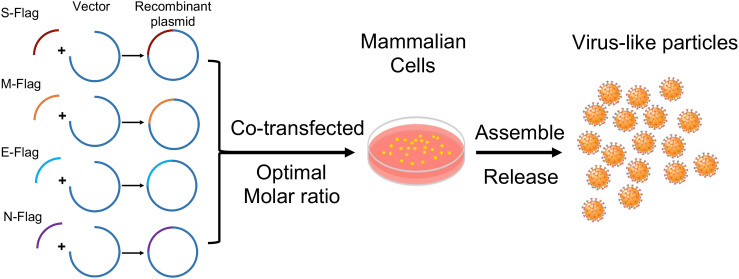
Schematic outline of SARS-CoV-2 VLPs constructions in mammalian expression system.

Cells were seeded into 6-well plate coated with Poly-D-lysine (Sigma Aldrich, St. Louis, MO, United States) 12 h before transfection. The plasmid constructs were transfected or co-transfected with lipofectamine 3000 (Invitrogen) and Opti-MEM reduced serum medium (Gibco) into HEK-293T cells and Vero E6 cells, respectively. For transfection of single plasmids, a total of 2.5 μg of each plasmid was applied individually. Co-transfection of double and triple plasmids was conducted with equal molar of each plasmids. Construction of SARS-CoV-2 VLPs was performed by co-transfecting cells with S, M, E, and N with molar ratio at 8:6:8:3 as shown in Figure 1. SARS-CoV-2 VLPs were harvested 48 h post transfection. VLPs were obtained from culture medium by centrifugation at 1,000 rpm for 10 min at 4°C, followed by a second centrifugation at 2,000 × *g* for 10 min at 4°C. Afterward, the collected supernatant was filtered through a 0.45 μm filter membrane (Millipore, Billerica, MA, United States), and then the filtrates were centrifuged with 20% sucrose at 21,000 × *g* for 7 h at 4°C. The final pelleted particles were recovered in ddH_2_O. Meanwhile, attached cells were washed with cold PBS, and then harvested and lysed with prechilled lysis buffer (50 mM Tris-HCl, pH 7.5, 150 mM NaCl, 1.0% NP-40, 0.1% sodium dodecyl sulfate, 5 mM EDTA, 1 mM Na_3_VO_4_, 1 mM NaF, supplemented with protease inhibitor cocktail). After centrifuge at 13,000 rpm for 15 min at 4°C, cell lysate was collected. Alternatively, VLP-containing pellets from 20% sucrose centrifugation were resuspended in TNE buffer [50 mM Tris-HCl, 100 mM NaCl, 0.5 mM EDTA (pH 7.4)], loaded on top of 20–60% discontinuous sucrose gradients and ultra-centrifuged for 3.5 h at 26,700 rpm. Nineteen fractions were then collected and analyzed by immunoblotting.

### Immunoblotting

The collected samples were applied to 4–20% SDS-PAGE. After SDS-PAGE separation and transfer, membrane blocking was performed with 3% BSA (Sigma) for 1 hr at room temperature. Primary antibody against flag (anti-DDDDK, MBL, Nagoya, Japan), against SARS-CoV-2 spike glycoprotein (1:1,000, Abcam, United States), against SARS-CoV-2 nucleoprotein/NP (1:1000, Sino Biological, Beijing, China), and secondary anti-Rabbit IgG (1:2000, Abcam) were applied before image capture. The expression of VLP proteins was developed by high sensitivity western chemiluminescent HRP substrate (Millipore) and visualized under ChemiDoc MP Imaging System (Bio-Rad, United Kingdom).

### Negative Staining of Electron Microscopy

For negative staining, collected VLPs were placed onto carbon-coated grids for 1 min and stained with phosphotungstic acid (Sigma) for 45 s. After air-dry overnight, the thin sections were observed and imaged with a transmission electron microscope (H7650, Hitachi, Japan) at 100 kV.

### Statistical Analysis

All experiments were repeated at least three times independently. All data are presented as mean ± standard deviations (SD) with Student’s *t*-test. The level of statistical significance was set for *p* < 0.05. Data and graphs were generated by GraphPad Prism 7.0, and figures were performed using Illustrator CC 2018 (Adobe).

## Results and Discussion

### Expression and Efficient Formation of SARS-CoV-2 VLPs in Mammalian Expression System

**FIGURE 2:**
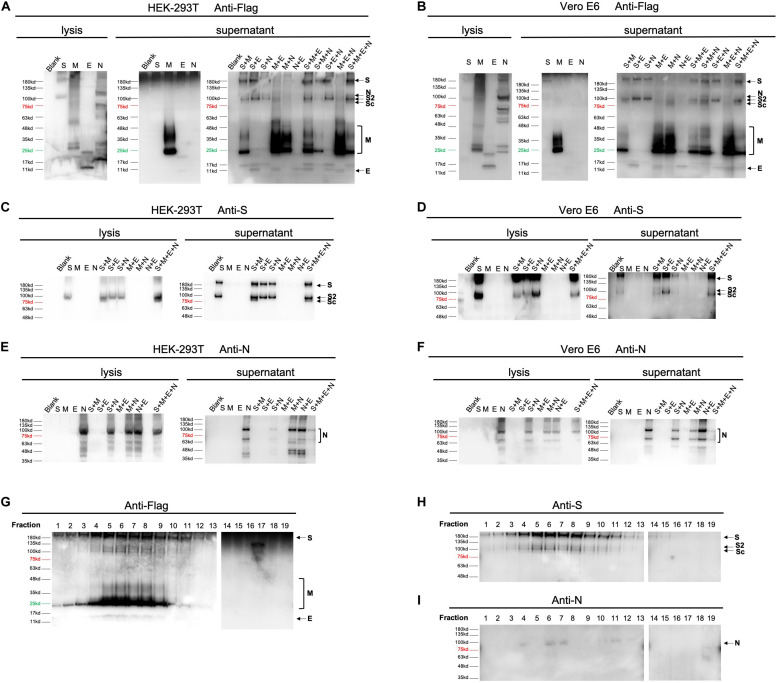
Expression of SARS-CoV-2 VLPs in mammalian expression system. Expression of four structural proteins analyzed using collected cell culture supernatant and cell lysate 48 h after transfection or co-transfection of plasmids encoding S, M, E, or N in both HEK-293T **(A,C,E)** and Vero E6 cells **(B,D,F)**. In single plasmid transfection, a total of 2.5 μg of each plasmid was applied to HEK-293T or Vero E6 cells individually. For double and triple plasmids co-transfection, S, M, E, or N plasmid was introduced at equal molar ratio. Construction of SARS-CoV-2 VLPs was performed by co-transfecting cells with S, M, E, and N with molar ratio at 8:6:8:3. The overall expression profile of four structural proteins was visualized using anti-flag antibody **(A,B)**. Anti-S **(C,D)** and anti-N antibodies **(E,F)** were additionally used to confirm expression of S and N, which could not be clearly distinguished by anti-Flag antibody. **(G–I)**, resuspended pellets from ultracentrifuged cell medium were then loaded on a 20–60% discontinuous sucrose gradient and subjected to fractions by ultracentrifugation. Nineteen fractions were collected (1–19, from lightest to heaviest). The nature of viral proteins associated with each fraction was determined by anti-Flag **(G)**, anti-S **(H)** and anti-N **(I)** antibodies. S, full-length spike protein; S2, membrane fusion subunit of spike protein; Sc, a cleaved membrane fusion subunit of spike protein. Protein marker and negative control (Blank) are shown as indicated.

To get better understanding of the secretory features of four SARS-CoV-2 structural proteins, we first transfected HEK-293T and Vero E6 cells with vectors expressing S, M, E, or N, respectively. As shown in Figures 2A,B, M protein was able to easily release into medium (supernatant) independent of other structural proteins 48 h post-transfection. E protein could also be secreted when expressed alone, but to a much less extend compared to M, indicating that M was an essential driver of VLP formation. Besides, S and N were least detectable in culture supernatant in the absence of other structural constituents when detecting with Flag antibody. Application of anti-S (Figures 2C,D) and anti-N antibody (Figures 2E,F) additionally confirmed expression of S and N, respectively.

To gain further insight into how S, M, E, and N may mutually be regulated in terms of protein egress, we then co-transfected HEK-293T and Vero E6 cells with equal molar of 2 out of 4 vectors (Figures 2A–F). Our data showed that in both cells, releasing of S could be enhanced by adding either M, or E, or N, suggesting that efficient formation of S-containing VLP could be driven by co-expression of any other proteins (Figures 2A–D). Interestingly, presence of M resulted in cleavage of the membrane fusion subunit of spike protein (Sc, Figures 2A–D). Moreover, secretion of M was further boosted when co-expressing M with E or N, but slightly suppressed in the presence of S (Figures 2A,B). This observation inferred that E and N played an additive role in M-based VLP egress. Unlike S and M protein, both E and N did not exhibit significantly altered secretory pattern when other elements co-existed, pinpointing that E and N were relatively independent in VLP construction.

Aiming to verify the correlation of proteins in double-transfection systems, we next explored the efficiency of protein release by combination of three expressing vectors. Consistent with pairwise relationships, addition of E to S-harboring systems (S + M and S + N) dramatically increased the amount of egressed proteins, confirming the supportive function of E. Moreover, incorporations of S into M-presenting conditions (M + E and M + N) resulted in restrained release of M, highlighting a limiting effect of S on M. In addition, supplementing M or N had no further effects on cells expressing any other two constituents (Figures 2A,B).

By using 20–60% discontinuous sucrose gradient (Figures 2G–I), we were able to cosediment four secreted structural proteins in the same fractions of sucrose gradient (fraction 6 and 7), suggesting that viral proteins are associated into VLPs.

Altogether, these data establish for the first time that M is the most critical protein that drives SARS-CoV-2 VLP egress. Presence of E may substantially potentiate the release efficiency of both M and S. Considering that viral infectivity depends highly on S, the minimal molecular requirement for an efficient assembly and egress of SARS-CoV-2 VLP is both M and E proteins.

### Morphological Evaluation of SARS-CoV-2 VLPs

**FIGURE 3:**
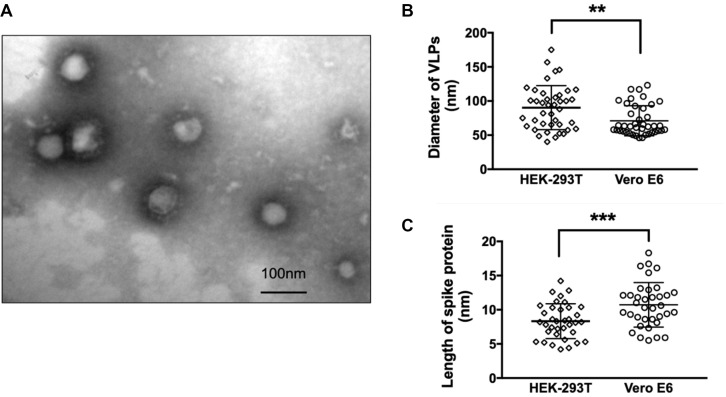
Morphological evaluation of SARS-CoV-2 VLPs. **(A)** TEM images of SARS-CoV-2 VLPs constructed from Vero E6 cells 48 h after co-transfection of S, M, E, and N plasmids with molar ratio at 8:6:8:3. Scale bar = 100 nm. **(B)** Quantification and comparison of diameters of SARS-CoV-2 VLP particles formed from both HEK-293T and Vero E6 cells. **(C)** Quantification and comparison of the length of spike proteins incorporated into SARS-CoV-2 VLP particles derived from both HEK-293T and Vero E6 cells. **p < 0.01; ***p < 0.005. Data were mean ± SD.

Though the potency of protein egress is stronger in HEK-293T cells compared to Vero E6 systems (Figures 2A,B), the morphology of SARS-CoV-2 VLP derived from Vero E6 cells (Figure 3A) was more stable and unified in relative to that of HEK-293T. In TEM images of SARS-CoV-2 VLPs from Vero E6 cells, we could easily identify particles displaying typical corona-like structure which is the signature of the optimal formation of spike trimers on SARS-CoV-2 envelope. Compared to SARS-CoV-2 VLPs constructed from Vero E6 cells, VLPs formed by HEK-293T were more variable both in shape and size. Specifically, the average diameter of SARS-CoV-2 VLPs from HEK-293T cells falls around 90.33 ± 32.45 nm, whereas those assembled in Vero E6 cells were smaller, showing about 71.02 ± 21.98 nm (Figure 3B). Apart from diameter, the length of spike protein from these two systems was remarkably distinctive, exhibiting 8.33 ± 2.55 nm in SARS-CoV-2 VLPs from HEK-293T cells, and 10.72 ± 3.253 nm in Vero E6 systems (Figure 3C). Hence, our data suggest that Vero E6 cells are more efficient in optimal formation of trimeric spikes.

## Conclusion

In the current study, we described the efficient construction of SARS-CoV-2 VLPs by plasmid-driven transfection of viral structural proteins in mammalian cells. By comparing expression induced by single, double or triple expressing vectors, we are able to demonstrate that M and E are basically required for efficient assembly and release of SARS-CoV-2 VLPs. Similar to other members in *coronaviridae* family, M protein is the most abundant envelope protein that drives other structural components to be packed into VLPs. Though E protein has also been implicated beneficial in viral morphogenesis and release, the mechanistic action of E remains unclarified. The S protein, which is responsible for receptor binding, membrane fusion and as targets of drug and vaccine development, and N protein which encapsidates viral genome into virions, do not seem to have indispensable roles in SARS-CoV-2 VLPs assembly. Our morphological evaluation of SARS-CoV-2 VLPs derived from Vero E6 cells further confirmed a high incorporation of S glycoprotein on the surface of VLPs. To the best of our knowledge, most current studies focus on pseudo-virus-based platform of SARS-CoV-2, a successful construction of SARS-CoV-2 VLPs via mammanlian expression system has not yet been reported. The approach in our study suggests that SARS-CoV-2 VLPs molecularly and morphologically resemble the native virion particles, which not only hold promise for virological research, but also present a potential vaccination for SARS-CoV-2.

## Data Availability Statement

All datasets presented in this study are included in the article/supplementary material.

## Author Contributions

NL, RX, and JL conceived and designed the experiments. RX and NL conducted the experiments and analyzed the data. NL, RX, MS, and PS wrote the manuscript. All authors contributed to the article and approved the submitted version.

## Conflict of Interest

The authors declare that the research was conducted in the absence of any commercial or financial relationships that could be construed as a potential conflict of interest.
