# Corrigendum: Construction of SARS-CoV-2 Virus-Like Particles by Mammalian Expression System

**DOI:** 10.3389/fbioe.2020.01026

**Published:** 2020-09-09

**Authors:** Ruodan Xu, Mingfei Shi, Jing Li, Ping Song, Ning Li

**Affiliations:** ^1^Institute of Basic Theory for Chinese Medicine, China Academy of Chinese Medical Sciences, Beijing, China; ^2^Dongzhimen Hospital, Beijing University of Chinese Medicine, Beijing, China; ^3^Guang'anmen Hospital, China Academy of Chinese Medical Sciences, Beijing, China

**Keywords:** COVID-19, SARS-CoV-2, virus-like particles, self-assemble, release

In the original article, there was an error in the Materials and Methods section, subsection Plasmid Construction and Molecular Cloning. The section should read as follows:

Human codon optimized sequences of genes encoding S, M, E and N structural proteins of SARS-CoV-2 with C-terminal FLAG tag (peptide sequence: DYKDDDDK) were synthesized by Genscript Biotechnology (Nanjing, China): the major structural S glycoprotein (Gen Bank: QHD43416.1), E protein (Gen Bank: QHD43418.1), M protein (Gen Bank: QHD43419.1) and N protein (Gen Bank: QHD43423.2). *NheI* and *NotI* (NEB, England BiolLabs, Beverly, MA, United States) restriction sites were placed at 5′ and 3′ ends, respectively. The four genes were cloned into the double *NheI* and *NotI* restriction sites of the expression vector pcDNA3.1. Then the transformation experiments were performed with chemically competent cells DH5α (TransGen Biotechnology, Beijing, China) using the heat shock method in the water bath at 42°C for 1 min, followed by shaking at 37°C for 45 min. After centrifugation at 2,800 × *g* for 3 min, the transformed cells were plated on LB plates containing 50 μg/ml ampicillin and the plates were inverted and incubated at 37°C overnight. The resistant single colony was picked and amplified in LB medium. The correct orientation of the insertions was examined by restriction enzyme analysis and the open reading frames of recombinant plasmids were verified by DNA sequencing.

The authors apologize for this error and state that this does not change the scientific conclusions of the article in any way. The original article has been updated.

